# Tissue Engineering of Canine Cartilage from Surgically Debrided Osteochondritis Dissecans Fragments

**DOI:** 10.1007/s10439-021-02897-7

**Published:** 2021-12-27

**Authors:** Natalia Vapniarsky, Lilia Moncada, Carissa Garrity, Alice Wong, Barbro Filliquist, Po-Yen Chou, Amy S. Kapatkin, Denis J. Marcellin-Little

**Affiliations:** 1 Department of Pathology, Microbiology, and Immunology, University of California, Davis, One Shields Ave., Davis, CA 95616 USA; 2 Department of Anatomy, Physiology and Cell Biology, School of Veterinary Medicine, University of California, Davis, 4206 VM3A, Davis, CA 95616 USA; 3 Department of Surgical and Radiological Sciences, School of Veterinary Medicine, University of California, Davis, 2112 Tupper Hall, Davis, CA 95616 USA

**Keywords:** Scaffold-free tissue-engineering, Self-assembly, Arthritis, Osteochondrosis, Thyroid hormone, Bioengineering

## Abstract

This study in dogs explored the feasibility of using cartilage fragments removed and discarded during routine palliative surgery for osteochondritis dissecans (OCD) as a source of primary chondrocytes for scaffold-free cartilage tissue-engineering. Primary chondrocytes were obtained from three OCD donors and one age-matched healthy articular cartilage (HAC) donor. After monolayer expansion of primary cells, a three-dimensional spherical suspension culture was implemented. Following this stage, cells were seeded at a high density into custom-made agarose molds that allowed for size and shape-specific constructs to be generated via a method of cellular self-assembling in a scaffold-free environment. Fifty-eight neocartilage constructs were tissue-engineered using this methodology. Neocartilage constructs and native cartilage from shoulder joint were subjected to histological, mechanical, and biochemical testing. OCD and HAC chondrocytes-sourced constructs had uniformly flat morphology and histology consistent with cartilage tissue. Constructs sourced from OCD chondrocytes were 1.5-times (32%) stiffer in compression and 1.3 times (23%) stronger in tension than constructs sourced from HAC chondrocytes and only 8.7-times (81%) less stiff in tension than native tissue. Constructs from both cell sources consistently had lower collagen content than native tissue (22.9%/dry weight [DW] for OCD and 4.1%/DW for HAC vs. 51.1%/DW native tissue). To improve the collagen content and mechanical properties of neocartilage, biological and mechanical stimuli, and thyroid hormone (tri-iodothyronine) were applied to the chondrocytes during the self-assembling stage in two separate studies. A 2.6-fold (62%) increase in compressive stiffness was detected with supplementation of biological stimuli alone and 5-fold (81%) increase with combined biological and mechanical stimuli at 20% strain. Application of thyroid hormone improved collagen content (1.7-times, 33%), tensile strength (1.8-times, 43%), and stiffness (1.3-times, 21%) of constructs, relative to untreated controls. Collectively, these data suggest that OCD chondrocytes can serve as a reliable cell source for cartilage tissue-engineering and that canine chondrocytes respond favorably to biological and mechanical stimuli that have been shown effective in chondrocytes from other animal species, including humans.

## Introduction

The very low regenerative capacity of cartilage combined with a high demand for the joint return to function invite exploration of new strategies for replacement and regeneration of this tissue.^37^ The most common therapies currently available for the repair of cartilage are microfracture, autologous chondrocyte implantation with or without matrix (MACI and ACI, respectively), and autologous or allogeneic osteochondral grafts.^17^ MACI and ACI are the only FDA-approved cellular therapies available to patients to date in the US. Although successful, these methods have shortcomings. For instance, microfracture results in the repair of a cartilage defect by fibrocartilage, which is less durable than hyaline articular cartilage.^17^ Techniques that involve autologously sourced cells (i.e., ACI, mosaicplasty) require the harvest of healthy cartilage from non-weight bearing areas of the joint. Ostensibly, the quantity of such cartilage is limited, and its harvest introduces donor-site morbidity in the patient.^10,17^ While harvesting cells from an allogeneic source can address these concerns, the use of allogenic cells increases the risk of disease transmission and immune response.^11^ The shortcomings of the current treatments invite investigation into novel strategies for cartilage regeneration and replacement.

Tissue-engineering (TE) is a process that uses biologically relevant cells, scaffolds, and extrinsic stimuli to create a tissue that can mimic the function of its native counterpart.^26^ The use of scaffolds in cartilage TE has been recently contested by the egression of the scaffold-free engineering method.^8,16^ During the scaffold-free TE process, chondrocytes are expanded and seeded at a high density into custom-made molds. At high-density seeding, chondrocytes are thought to recapitulate the processes of embryonic joint development known as mesenchymal condensation.^8^ Following seeding, chondrocytes generate extracellular matrix constituents and yield neo tissue with mechanical and biological properties akin to native cartilage.^8^ The advantage of this novel method is in eliminating limitations and risks associated with the use of scaffolds.^17^

Cell source for cartilage TE continues to be a topic of debate and exploration. Mesenchymal stromal cells (MSCs) have been explored as a cell source for cartilage TE due to their capacity of multilineage differentiation.^2,44,45,54^ While the multipotency of MSCs would allow for chondrogenic differentiation *in vitro*, heterogeneity of MSC and their tendency to spontaneously differentiate pose challenges when using this cell source.^50^ This cell source's unpredictability makes it a less attractive candidate for cartilage TE than the terminally-differentiated chondrocyte. Clinical observations indicate that OCD fragments that are usually removed and discarded following surgery contain viable chondrocytes.^3^ Chondrocytes isolated from human OCD fragments have significantly more type X collagen mRNA and extended fibrous degeneration on the surface of the OCD cartilage.^3^ However, the expression of matrix molecules, cell viability, growth factors, and IL-1 are similar to the normal articular cartilage.^3^ A study in horses determined that chondrocytes harvested from OCD cartilage can undergo limited *in vitro* chondrogenesis, despite having perturbations of cell phenotype in vivo.^12^ These latter reports led to the conception of this study; using surgical fragments of cartilage removed during routine OCD surgery in dogs as a cell source for cartilage TE with a scaffold-free approach.

Osteochondritis Dissecans (OCD) is a developmental joint disease that has been described in both humans and veterinary species.^35^ Importantly,^51^ the spontaneously occurring nature of this disorder in animals presents an opportunity for translational research. While the etiology of OCD is still being debated, the outcome of this condition is focal cartilage necrosis that leads to the formation of a ‘blister’ or a cleft and eventual detachment of a cartilage flap from subchondral bone.^40^ OCD in dogs is most commonly diagnosed in the shoulder, elbow, stifle, and tarsal joints; involvement of more than one joint is common.^39^ The cartilage loss leads to mechanical and biochemical imbalances in the joint that culminate in osteoarthritis (OA).^46^

The study's first objective was to determine whether OCD fragment-sourced chondrocytes (OCD-chon) can serve as a reliable cell source for scaffold-free cartilage TE. We compared the biomechanical properties of TE neocartilage constructs sourced from OCD-chon and healthy articular chondrocytes (HAC-chon) to native tissue. We postulated that the functional properties of HAC-chon-derived constructs would surpass those of OCD-chon-derived constructs. The second objective was to investigate whether OCD chondrocytes respond to biological and mechanical stimuli previously reported to be effective on chondrocytes from other animal species and humans.^21,25,30,33^ We performed a full-factorial study exploring the effects of the application of biological (transforming growth factor beta -1 (TGF-β1), chondroitinase ABC (C-ABC), and Lysyl Oxidase L2 (LOXL2), mechanical (static compression), and a combination of biological and mechanical stimuli on the functional properties of OCD-chon-sourced scaffold-free TE neocartilage constructs. From previous studies that explored these biological factors on mammalian chondrocytes, it is known that TGF-β1 is inducing anabolic effects on chondrocytes that are manifested by extracellular matrix synthesis.^13,23^ C-ABC treatment of neocartilage constructs results in depletion of chondroitin and other glycosaminoglycans from the extracellular matrix, which presumably leads to closer opposition of collagen fibrils.^32^ LoxL2, in turn, induces pyridinoline cross-linking between the collagen fibers resulting in an overall increase in tensile strength and stiffness of TE neocartilages.^31,34^ We hypothesized that the supplementation of both biological and mechanical stimuli would have additive or synergistic effects on the functional properties of TE neocartilage.

Literature instructs on the positive effects of tri-iodothyronine hormone (T3) or thyroxine (T4) supplementation on collagen synthesis during cartilage manufacturing.^28,52^ These studies detected an increase in COL2A1 and biglycan (BGN) gene expression.^30^ Also, it is reported that thyroid hormone increases insulin growth factor 1 (IGF-1) expression on chondrocytes, stabilizing chondrogenic potential, stimulating SOX9 expression, and promoting molecular interactions between Erk and SOX 9 transcription factors.^47^ Since our experiments consistently demonstrated lower than native tissue collagen content in the constructs sourced from OCD- or HAC-chons, we explored if canine HAC-chon respond to thyroid hormone supplementation by increased collagen deposition (third objective). We hypothesized that T3 supplementation without mechanical stimuli would increase collagen content and consequentially improve TE neocartilage constructs tensile properties.

## Materials and Methods

### Study Design

**Figure 1:**
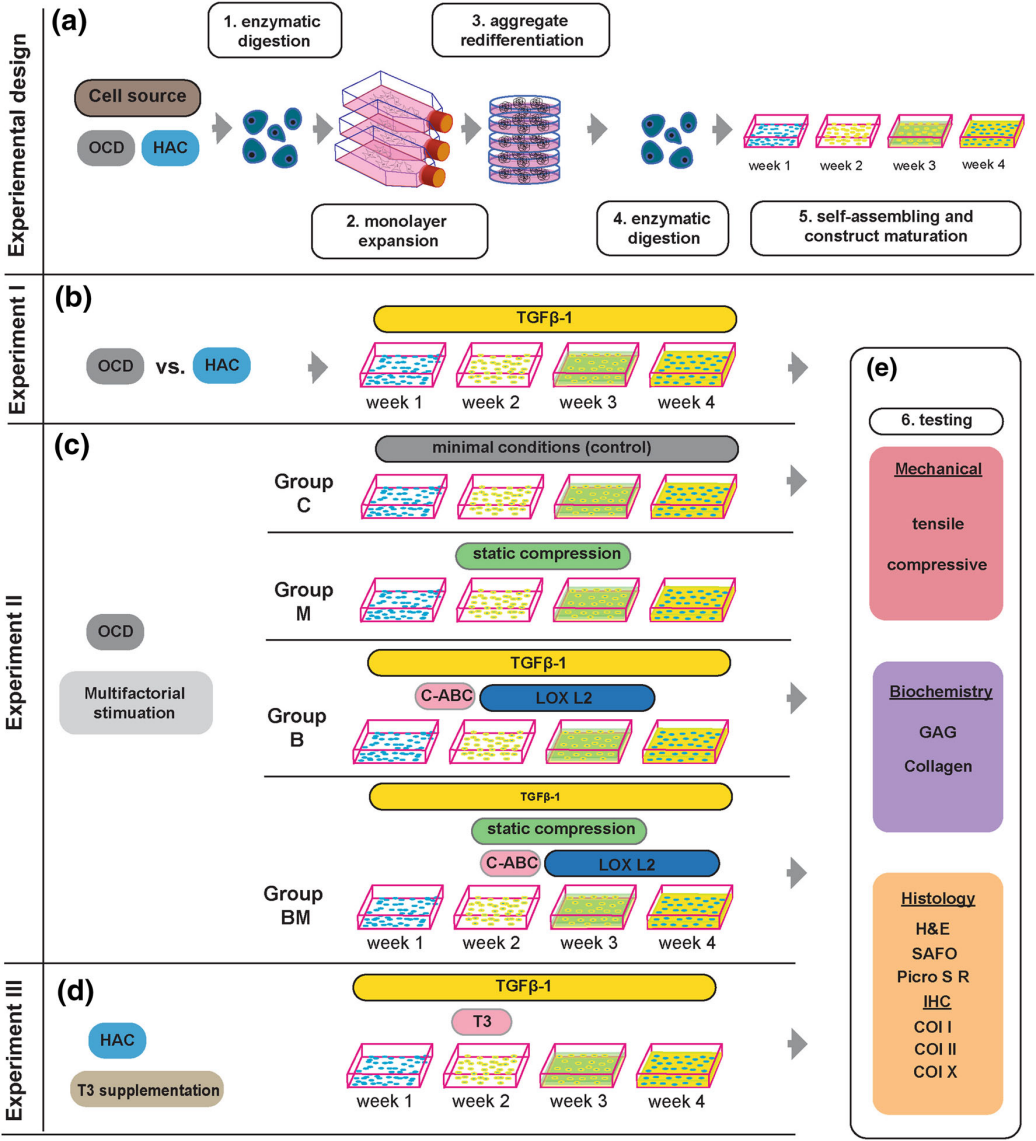
The study consists of three consecutive experiments that follow the same pattern - starting with chondrocyte harvest, processing, expansion, and culminating in construct generation (**A 1-5**). **A1** Chondrocytes are liberated from OCD flaps or healthy articular cartilage (HAC) ECM via enzymatic digestion with collagenase. **A2** Monolayer expansion in chondrogenic expansion media with the addition of growth factors yields millions of chondrocytes, but during this expansion, chondrocytes dedifferentiate into inferior fibroblast-like phenotype. **A3** A 3D spherical suspension culture (aggregate redifferentiation) reverts the cells to their chondrogenic phenotype. **A4** Following second enzymatic digestion, the cells are individualized in suspension. **A5** High-density seeding into custom-made agarose molds allows the process of self-assembling to yield neocartilage constructs without using exogenous scaffolds. The mold defines the size- and shape of cartilage constructs. A variety of biological and mechanical stimuli can be applied during this stage, mimicking the biological milieu. **B** Experiment I. Chondrocytes isolated from OCD surgical fragments (OCD-chon) or healthy articular cartilage (HAC-chon) are processed as described in A. The TGF-β1 supplementation is provided during the construct self-assembling and maturation phases. **C** Experiment II. During the maturation-phase, OCD-chon-sourced constructs are divided into four treatment groups that receive no stimulation (Group C = control), biological stimulation (TGF-β1, C-ABC, LOXL2 (Group B=Bio), mechanical stimulation (Group M = Mech), or a combination of biological and mechanical stimuli (Group BM= Biomech). **D** Experiment III. The tri-iodothyronine (T3) treatment is implemented during the first two weeks of the HAC-chon-sourced construct maturation phase. **E** All experiments. Upon maturation, TE neocartilage constructs and native tissue are subjected to biochemical, mechanical, histological, and immunohistochemical analyses to access and compare the functional properties of neotissue and to compare to its native counterpart. Abbreviation: IHC = immunohistochemistry, GAG = glycosaminoglycan.

The study consisted of three consequent experiments that followed the same protocol for chondrocyte monolayer expansion, aggregate culture, and high-density seeding (construct maturation phase/self-assembly). The three-dimensional spherical suspension culture was implemented (aggregate redifferentiation) to overcome cell dedifferentiation after a monolayer expansion (Fig. 1a).^19^ After completing the aggregate culture, cells were disassociated into a single cell suspension and seeded at a high-density (27,000 cells/*µ*l) into custom-made agarose wells using a scaffold-free, self-assembling method previously described.^19^ *In experiment I*, OCD-chon-derived constructs were compared to HAC-chon-derived constructs (Fig. 1b). *In experiment II*, OCD-chon-derived constructs were subjected to different treatments during the construct maturation phase, including no treatment, biological, mechanical, biological and mechanical stimulation (Fig. 1c). *In experiment III*, HAC-chon-derived constructs were treated with triiodothyronine (T3) during the construct maturation phase. An untreated group of the same cell source was used as a control (Fig. 1d). No mechanical or biological stimuli were applied in this experiment except TGFβ-1 throughout the culture duration. In all experiments, following the construct maturation phase, the tissue-engineered constructs were subjected to histological, mechanical, and biochemical testing (Fig. 1e).

### Tissue Collection

Osteochondritis Dissecans (OCD) fragments were collected during routine OCD surgical removal from the shoulder at the Veterinary Teaching Hospital (VMTH) at the University of California, Davis (UCD). Three OCD fragments from age-matched donors were included in the study (1-year-old Border Collie; 1-year-old German Pointer; 1-year-old Old English Sheepdog). Healthy articular chondrocytes (HAC) were collected from a limb of a 3-year-old, Shiba Inu that had a limb amputated due to a traumatic joint luxation and degloving injury. The patient’s amputated limb was released for unrestricted research use. Articular cartilage was processed as described below for OCD cartilage. Native articular cartilage specimens were collected from humeral heads of six deceased dogs released for unrestricted use. Patient information was limited to the fact that the dogs were euthanized for reasons other than musculoskeletal disease, and their median age was 3 years.

### Cell Isolation

OCD cartilage fragments digested in 2 mg/mL collagenase solution (Worthington Biochemical Corp. 290 active units/mg DW) supplemented with 30 *µ*L/mL (3% v/v) fetal bovine serum (FBS) (Atlanta Biologicals) for 18 hr. at 37°C, 5% CO_2_ with orbital rotation at 60 RPM. HAC-chondrocytes were obtained from the articular surface of the humeral head by transverse scoring of the articular surface followed by horizontal scoring and processed as described above for OCD cartilage. A cell count and viability were obtained using a hemocytometer for all specimens using Trypan blue vital staining. Chondrocytes that were not immediately used for expansion were cryopreserved in liquid nitrogen in 90% FBS and 10% Dimethyl Sulfoxide (DMSO) (Corning^®^ DMSO, Mediatech Inc.) until further use.

### Chondrocyte Expansion in Monolayer

OCD and HAC chondrocytes were seeded into cell culture flasks at 1.3 x 10^4^/cm^2^ and grown until 80% confluency in expansion media. The composition of expansion media is provided in detail in Table 1. The cells were passaged 2–3 times until a sufficient number of cells was reached to proceed to an aggregate redifferentiation phase. The media was replaced every 3–4 days or with every passage. For passaging, cells were lifted up following 10min digestion at 37°C with full strength Trypsin (Gibco 0.05% Trypsin-EDTA 1X, Cat# 25300-054), spun down at 400 RPM and further digested in 0.2% collagenase solution supplemented with 30 *µ*L/mL (3% v/v) FBS for 30–45 min at 37°C to remove any residual ECM that is produced by chondrocytes during monolayer expansion.

**Table 1:** Culture media formulations.

Component	Manufacturer/catalog #	Expansion media	Chondrogenic media
DMEM	Gibco 10566-016	Full strength	Full strength
Pen/strep amphotericin B	BioWhitaker 17-745E	10 mg/mL	10 mg/mL
MEM non-essential amino acids	Gibco 11140-050	10 mg/mL	10 mg/mL
Insulin/human transferrin/selenium (ITS+) premix	Corning 0354352	10 mg/mL	10 mg/mL
Sodium pyruvate	Thermo P2256-25g	100 μg/mL	100 μg/mL
L-Ascorbate-2-phosphate	Sigma Aldrich A8960-5g	50 μg/mL	50 μg/mL
L-proline	Sigma Aldrich P0380-100g	40 *µ*g/mL	40 *µ*g/mL
Dexamethasone	Sigma Aldrich D4902-25mg	0.0104 mg/mL	0.104 mg/mL
Fetal bovine serum	Atlanta biologicals S11550	100 *µ*L/mL	NA
Human recombinant TGF beta 1	Pepro Tech 100-21 – 10 *µ*g	1 ng/mL	10 ng/mL
Human recombinant FGF	Pepro Tech 100-18B – 100 *µ*g	5 ng/mL	NA
Human recombinant PDGF	Pepro Tech 100-14B	10 ng/mL	NA

After each passage, the cell suspension was assessed for viability with Trypan blue vital staining. Of note, during the expansion, the growth factors that were applied were recombinant human growth factors. Although not specific for canine species, these factors were proven to be effective on chondrocytes from bovine, ovine, and porcine species at the effective concentrations used here.^18,22,25,29^ Additionally, a BLAST search and comparison was performed to determine sequence similarity between dog and human growth factors TGF-β1, FGF2, and PDGF. Each growth factor's protein sequence was obtained through an individual internet search (www. Uniprot.org). Once the sequence was obtained, it was plugged into the BLAST Global Protein Alignment software to determine the similarity percentage between the two species. The similarity of dog and human protein sequences of PDGF, TGF-β1, and FGF2 were 93%, 94%, and 74%, respectively. The degree of similarity between dogs' and humans' growth factor proteins suggests that using human-derived growth factors would be acceptable.

### Population Doubling and Real-Time PCR Gene Analyses

Population doubling times were determined according to the previously described protocol and the formula.^42,49^ Briefly, at each passage, cells were seeded at a constant density of 40,000 cells/cm^2^ and grown until 90% confluency. The duration between passages was recorded for a total of ten passages.

For the real-time, quantitative PCR analysis, P0 cells from OCD and HAC cartilage were selected. Total RNA was extracted from cells using RNeasy Mini Kit (Qiagen, USA), and 1000 ng of total RNA was reverse-transcribed using the QuantiNova Reverse Transcription Kit (Qiagen, USA). Primer sets are presented in Table 2. PCR reactions were carried out using the QuantiNova SYBR Green PCR master mix (Qiagen). Cycle conditions were denaturation at 95 °C for 2 min, and 40 cycles of denaturation at 95°C for 5 s, annealing at 60 °C for 15 s.

**Table 2:** Real-time PCR primer sets.

Gene	Forward primer	Reverse primer
Col1A2	GCTTGCAGTAACTTCGTGC	TACCATCGTCACCATCTCTGC
Col2A1	GGTCCTCAAGGCAAAGTCG	AGGGAAACCCATGACACC
Aggrecan	ACTTCCGCTGGTCAGATGGA	TCTCGTGCCAGATCATCACC
Col X	TTTCTCCTACCACGTGCATG	GAAGCCTGATCCAGGTAGCC

### Aggregate Redifferentiation

Following sufficient expansion, OCD and HAC chondrocytes were suspended in chondrogenic media and seeded into 90 mm Petri plates coated with 20mg/ml agarose at a density of 1 million cells/mL in a total volume of 15–20 mL. Chondrogenic media formulation is provided in Table 1. For the entire duration of aggregate culture, chondrogenic media was supplemented with 10 ng/mL TGF-β1. The cells were cultured at 37°C in 5% CO_2_ with orbital rotation at 60 RPM for 24 hr. After 24 h, plates were maintained under the same conditions but static. The media was replenished every 3–4 days for a total of 10 days. On the 11th day, aggregates were examined under a microscope and digested to single-cell suspension with 15 mL/plate of 0.05% Trypsin for 45 min in 37°C followed by digestion in 15 mL of 2 mg/mL collagenase solution supplemented with 30 *µ*L/mL (3% v/v) FBS for 90 min. Following an additional wash step, the cell count was obtained, and cell viability was assessed with Trypan blue vital staining.

### Construct Seeding (Self-Assembling)

OCD-chon and HAC-chon constructs were seeded at 8 million cells in a seeding volume of 300 *µ*L of chondrogenic media into custom-made 7mm x 12mm agarose molds. After 4 hr., constructs were topped off with an additional 2 mL chondrogenic media supplemented with reagents appropriate for each experimental treatment group (Fig. 1). The constructs were provided with chondrogenic media that was replenished every other day for the duration of four weeks, at which point construct development was stopped. All TE constructs from experiments I-III were subjected to the same testing protocols on day 28 of self-assembling.

*In experiment* I, the OCD-chon and HAC-chon constructs were provided with chondrogenic media supplemented with 10 ng/mL TGF-β1 throughout the four weeks of the self-assembling process.

*In experiment II*, the control group (**C group**) constructs were provided with plain chondrogenic media. Constructs in the biological group (**B group**) were provided with chondrogenic media supplemented with 10 ng/mL TGF-β1 throughout, C-ABC on day 7 for 4 hr., and LOX L2 on days 14–28. This treatment regimen was optimized before.^25^ The C-ABC (Sigma C3667) treatment was delivered at a concentration of 1.54 units/mL in chondrogenic media. Each construct was incubated in 1.4 mL of C-ABC for 4 hr. at 37°C, 5% CO_2_. C-ABC was neutralized *via* incubation in chondrogenic media supplemented with 1mM zinc sulfate for 10 min. Constructs were then washed two more times with chondrogenic media to ensure the complete removal of C-ABC. The LOXL2 protein (SignalChem 20 *µ*g in 200 *µ*L, Cat# 262-31-G-100) was supplemented at 0.15 *µ*g/mL with the addition of hydroxylysine at 0.146 *µ*g/mL (Sigma Aldrich), and copper sulfate at 1.6 *µ*g/mL (Sigma Aldrich) at each media change. Constructs in the mechanical group (**M group**) were provided with plain chondrogenic media, and on day 10 of self-assembling, they were transferred into static compression wells (custom-made from 2% agarose). A static weight of 45 g (equivalent to 0.441 N) was then applied to each construct and left on for a total of 4 days (days 10–14). Constructs in the biomechanical group (**BM group**) received a combination of biological and mechanical stimuli, as described above.

*In experiment III*, constructs in T3 and control groups were provided with chondrogenic media supplemented with 10 ng/mL TGF-β1 throughout the duration of the self-assembling period of four weeks. For the T3 treatment group, the media was additionally supplemented with 25 ng/mL of tri-iodothyronine^28^ (Sigma Aldrich Lot# BCBW8768) on days 1–14 with each media change, every other day.

### Histology

Tissues were formalin-fixed and embedded in paraffin. Hematoxylin and eosin (H&E), Safranin-O with Fast Green counterstain (SAFO/FG), and Picosirius Red (PSR) stains were applied on 5 *µ*m sections according to previously published standard protocols.^6^ The images were obtained using CellSense/Olympus software and Olympus microscope (Olympus Optical Co., LTD, model: BX40F4) equipped with a color camera (Olympus DP72). Cell counts and cell diameters were measured using ImageJ software (ImageJ Public License, imagj.nih.gov, Version 1.51 23). Cell viability within the neocartilage constructs was evaluated by a board-certified histopathologist (NV).

### Biochemical Testing

Representative portions of all TE constructs from experiments I-III and native tissue samples were preserved frozen at − 80°C until testing. The wet and dry weights were obtained before and after 24 h. lyophilization, respectively. Lyophilized samples were digested in papain solution (125 *µ*g/mL Papain + 5 mM *N*-Acetyl-l-Cysteine + 5 mM EDTA (anhydrous) + 100 mM Phosphate Buffer) at 1 *µ*l of digestion solution per 1 *µ*g of sample dry weight at 60°C for 18 h. The collagen content was assessed by OH-Proline Assay (Sigma Aldrich) following the manufacturer’s instructions. The collagen content was then calculated based on a bovine collagen standard curve. The concentration of sulfated glycosaminoglycans in each sample was determined *via* a GAG Blyscan Assay (Bicolor Life-Science Assays) based on 1, 9-dimethyl methyl blue binding, following manufacturer instructions.

#### Mechanical Testing

For the tensile testing of native cartilage, 10 mm in diameter circular articular cartilage explants were trimmed off the subchondral bone. Dumbbell-shaped samples were cut from native tissue or TE constructs while maintaining a length to width of approximately 4:1. Each dumbbell-shaped sample was photographed. Thickness and width were measured and recorded using imaging software (ImageJ, U. S. National Institutes of Health, Bethesda, Maryland). A uniaxial tensile test was performed on a material testing machine (Instron 5565, Norwood, MA). The samples were elongated at a strain rate of 1%-gauge length per second. Stress/strain curves were generated from the load-displacement data. The load, elongation, and specimen geometry (width and thickness) were loaded into analysis software (Matlab, MathWorks, Natick, MA) and analyzed with a custom program that plots load normalized to cross-sectional area and strain. The output was used to identify the linear portion of the curve to determine tensile stiffness (Young’s modulus) and the ultimate tensile strength (UTS) point.

For compressive mechanical testing, flat, 3mm in diameter specimens were obtained from native and TE samples using a punch-biopsy knife. For the duration of compressive testing, samples were submerged in PBS at room temperature. A uniaxial, unconfined stress-relaxation testing was carried out on Instron 5565 (Norwood, MA). Samples were strained to 10% and 20% sequentially and allowed to relax for 600 and 1200s, respectively. The instantaneous (Ei) and relaxation moduli (Er) were calculated using a custom Matlab program upon fitting the data to a Kelvin solid viscoelastic model at each strain level.^1^

#### Immunohistochemical Analyses

Unstained sections of paraffin-embedded tissues were deparaffinized through submersion in xylene (Histological Grade Xylenes, Lot#161051, Fisher Chemical) three consecutive times for 4 min. Next, sections were rehydrated through submersion in decreasing concentrations of ethanol at (100%, 95%, and 75%) for 4 min each. Endogenous peroxidases were quenched by soaking the sections in 3% hydrogen peroxide solution in methanol for 30 min at room temperature (RT). Sections were then washed in PBS with 0.2% Tween. Approximately 600 *µ*l of antigen retrieval agent comprised of 0.4% pepsin was applied to each tissue section followed by incubation at 37°C for 20 min. A blocking buffer consisting of 5% goat serum in PBS was then applied to each tissue section and left at room temperature for 30 min. The primary antibody was then added at optimized concentrations for 1 hr. at room temperature (Table 3). After washing in PBS + Tween, the corresponding secondary antibodies (anti-rabbit, 1:1000) were added and washed off after 30 min. The ABC reagent (Vectastain Elite ABC Universal Kit, Ref# PK-6200, Vector Laboratories, Burlingame, CA) was applied and maintained for 30 min to ensure a strong streptavidin-biotin complex formation. The color was developed with the addition of NovaRed reagent (Vector Laboratories) for 5 min. Samples were dehydrated again in increasing concentrations of ethanol, counterstained with ½ strength Hematoxylin solution, and mounted using a Permount reagent (Vector Laboratories) Images were obtained using an Olympus photomicroscope with CellSense software, as described above.

**Table 3:** Antibodies and protocol information for IHC on native tissue and TE constructs.

Antibody name	Clone #	Dilution ratio	Producer	Antigen retrieval reagent	Host species	Product /Cat #
COL1A2	Polyclonal	1:300	*Lifespan BioSciences*	Pepsin 0.4%	Rabbit	LS-C35202
COL2	Polyclonal	1:250	*Abcam*	Pepsin 0.4%	Rabbit	AB34712
COL10	Polyclonal	1:100	*Biorbyt*	Pepsin 0.4%	Rabbit	A0714

#### Donor Comparison

Because chondrocytes from several OCD donors were assessed in this study, we addressed the donor as a biological variable. To do so, biochemical and mechanical properties of neocartilages manufactured with identical methods between two OCD donors were plotted side by side.

#### Statistical Analyses

For *experiment I*, an unpaired, two-tailed students’ *t* test was performed to explore the difference between biochemical and mechanical properties of OCD and HAC-sourced constructs. Each group represented a set of samples obtained from a single donor. In *experiment II*, due to low n per group, the normal distribution could not be demonstrated. Therefore, a non-parametric test (Kruskal-Wallis) with Dunn’s multiple comparisons test was used to detect significant differences in biochemical content or mechanical properties between the groups. In *experiment III*, an unpaired, two-tailed students’ *t* test was used to analyze differences in biochemical content or mechanical properties between two treatment groups. For comparison between donors, an unpaired two-tailed students’ *t* test was applied. For all experiments, a *p* value of < 0.05 established a significant difference.

## Results

### Population Doubling and Gene Expression of OCD and HAC Chondrocytes

**Figure 2:**
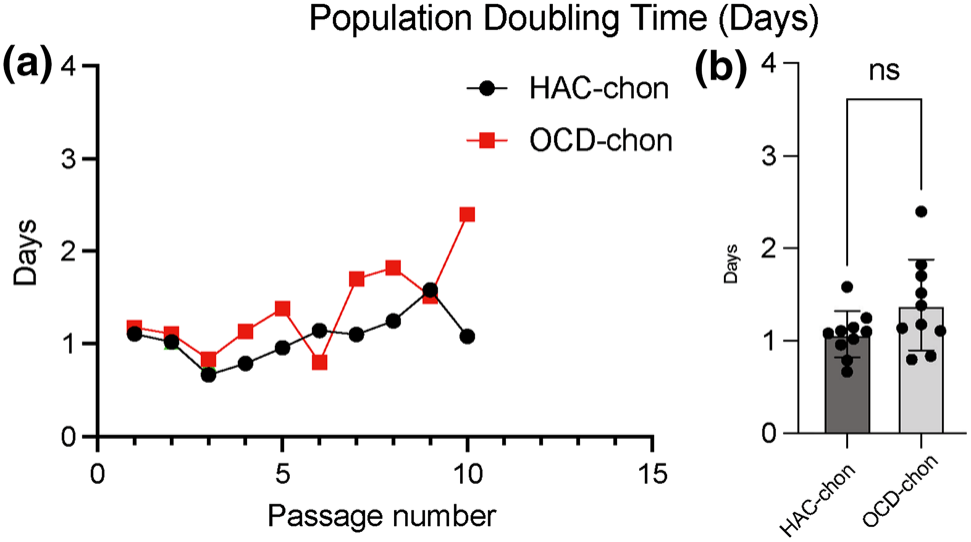
Population doubling times of HAC-chon and OCD-chon expanded in monolayer up to passage ten. **A** linear presentation of population doubling in days across ten passages. **B** Averaged population doubling times. *Ns* no significant difference.

The weight of OCD fragments ranged 28.2–47.3 mg. A total of 58 cartilage constructs were tissue-engineered; 28 were sourced from healthy articular cartilage (HAC-chon) and 30 from OCD fragments (OCD-chon). OCD chondrocytes grew well in monolayer and had a comparable population doubling times to HAC chondrocytes (Fig. 2).

**Figure 3:**
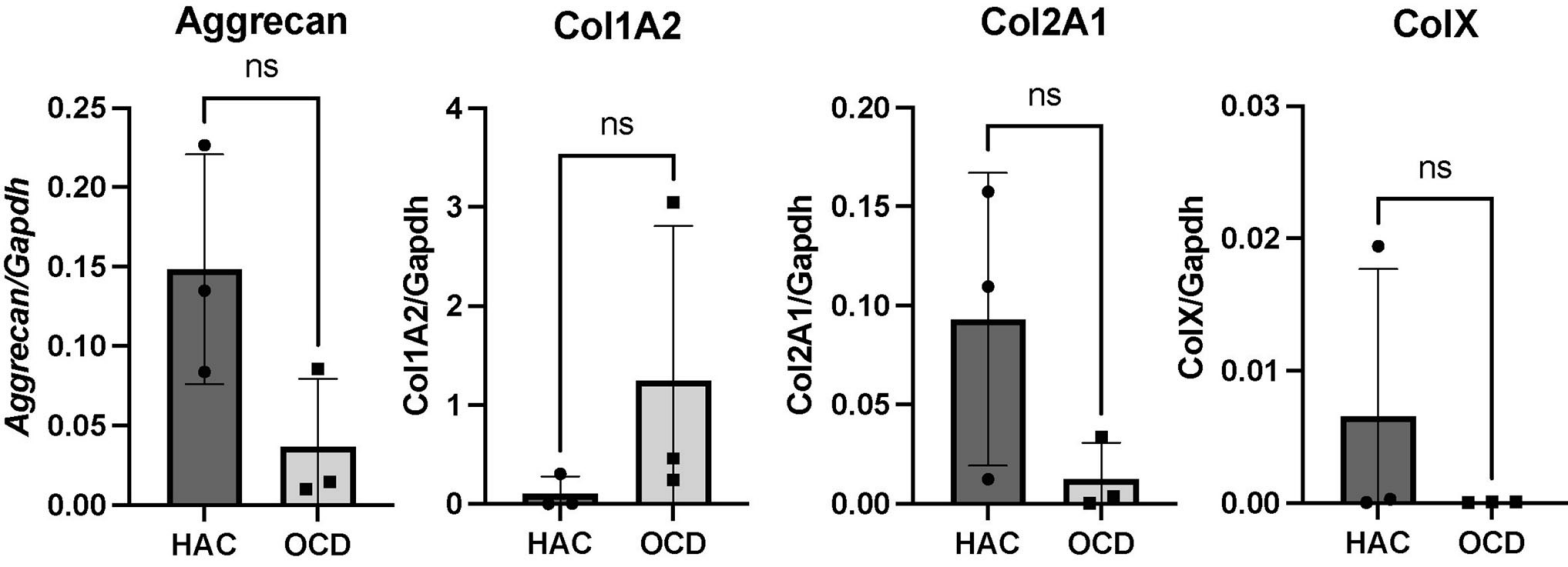
Real-time PCR analyses of OCD chondrocytes and healthy articular chondrocytes. Three OCD and HAC cartage donors were analyzed for expression of Aggrecan, Collagen type I (Col1A2); Collagen type II (Col2A1); and Collagen type X (ColX). *Ns* no statistically significant difference. The CT values were normalized to GAPDH housekeeping gene. All assays were performed in triplicates.

We detected no significant difference in Aggrecan, Col I, Col II, and Col X gene expression between OCD and HAC chondrocytes at P0 (Fig. 3).

### Macroscopic Morphology and Dimensions of Neocartilage Constructs

**Figure 4:**
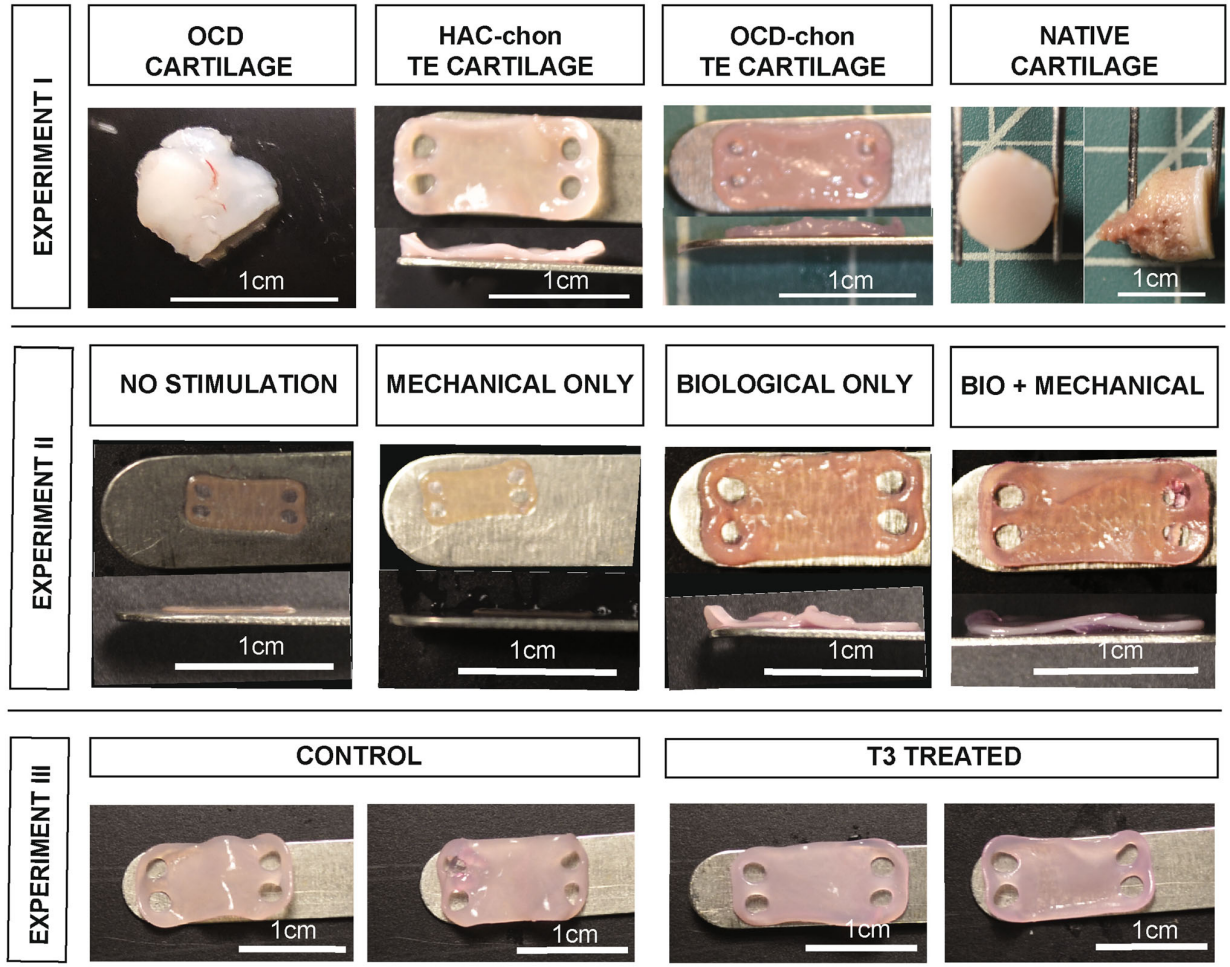
Gross morphology of OCD flap, native tissue, and TE neocartilage constructs from Experiments I-III. Note small and thin morphology of the constructs in no stimulation (C) and mechanical stimulation only (M) groups, Experiment II. Size bar = 1 cm.

Gross morphology of OCD- and HAC-chon-sourced neocartilage constructs in *experiment I* was smooth, flat, and hyaline-like (Fig. 4) with average dimensions of 6.0 ± 0.05 mm x 11.6 ± 0.4mm x 0.7 ± 0.08 mm and 7.0 ± 2.3mm x 16.5 ± 4.7 mm x 0.7 ± 0.3 mm, respectively. The average thickness of native articular cartilage samples was 0.56 mm. For the sake of consistency, all samples were obtained from the approximately same region in the joint (middle portion of the articulating surface).

In *experiment II*, the lack of mechanical and biological stimulation in the control group (minimal culture conditions, no biological or mechanical stimulation) resulted in very poor quality of constructs, and only three were suitable for testing. Similarly, mechanical stimulation alone yielded equally poor-quality constructs, and only two were available for evaluation. There were nine testable constructs in **B** (biological stimulation only) and six in **BM** (biological and mechanical stimulation) groups. The gross morphology of constructs differed notably between treatment groups. (Fig. 4). Although uniformly flat and smooth, constructs in the **C** (control, no stimulation) and **M** (mechanical stimulation only) groups were significantly smaller and thinner than constructs in B and BM groups. The length of the constructs in C, M, B, and BM groups was 6.9 ± 0.1 mm, 8.0 ± 1.3 mm, 14.9 ± 2.2 mm, and 15.3 ± 2.3 mm, respectively. The width was 3.0 ± 0.1 mm, 3.4 ± 0.5 mm, 7.0 ± 0.8 mm, and 7.1 ± 0.9 mm and the thickness was 0.2 ± 0.06 mm, 0.16 ± 0.4 mm, 0.6 ± 0.2 mm, and 0.6 ± 0.2 mm, respectively. Application of mechanical stimulation during construct maturation resulted in a slight increase in construct size relative to the control group. Albeit, the application of biological and combined biological and mechanical stimuli resulted in a significant increase in length, width, and thickness of constructs relative to the control group.

In *experiment III*, T3-treated constructs (T3 group) were overall similar morphologically to the control (no T3 supplementation) constructs (C group) (Fig. 4). The average dimensions of construct in C and T3 group were 14.4 ± 0.3 mm x 7.4 ± 0.2mm x 0.5 ± 0.1 mm and 15.4 ± 0.5 mm x 7.4 ± 0.2 mm x 0.5 ± 0.1 mm, respectively, with no significant difference between groups.

### Histology

**Figure 5:**
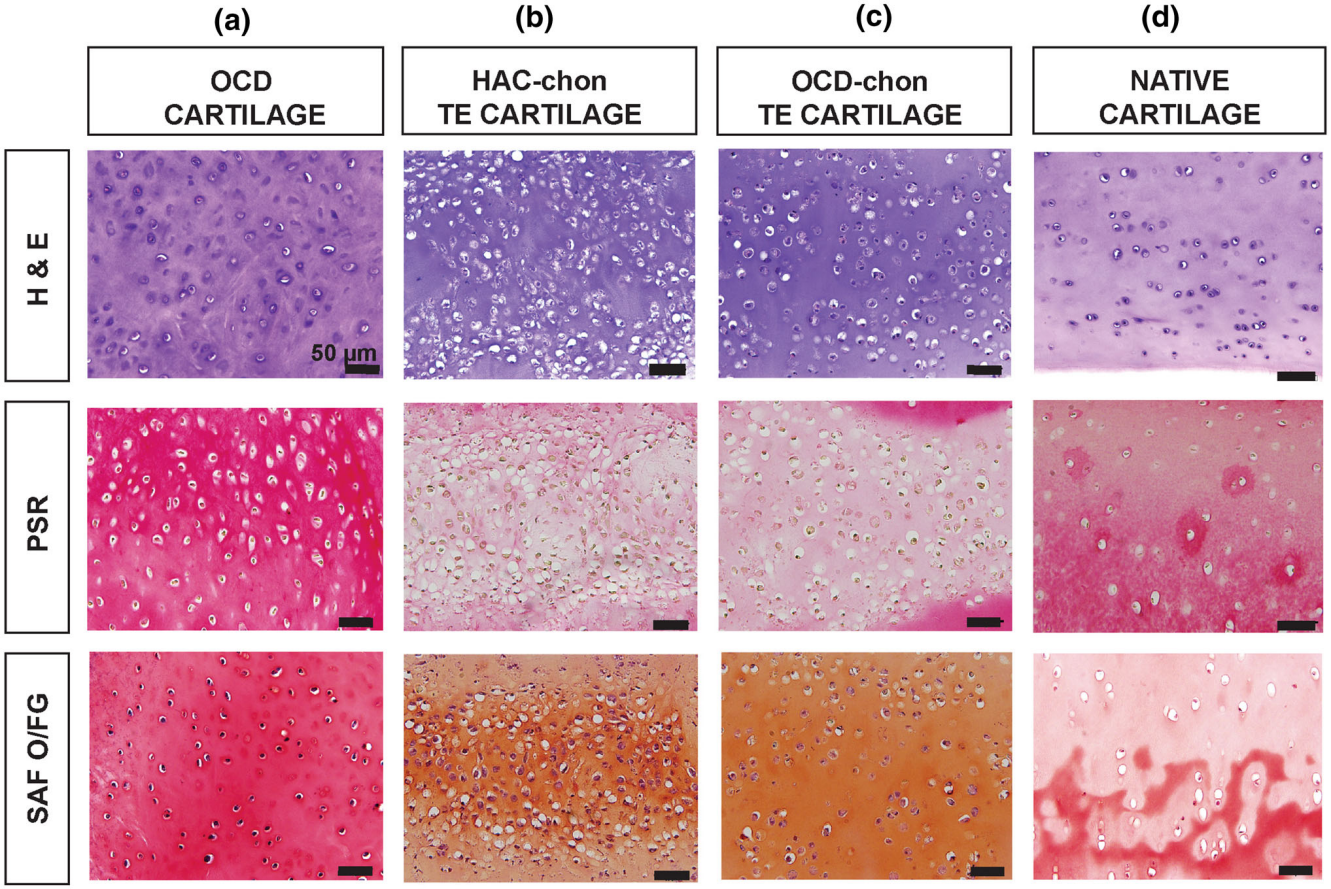
Histology of TE neocartilage constructs in comparison to native articular cartilage and OCD flap, Experiment I. H & E = Hematoxylin and Eosin stain, PSR = Picrosirius Red, SAF O/FG = Safranin O/Fast green counterstain. Size bars = 50 *µ*m.

Histological examination of OCD surgical fragments suggested the presence of viable chondrocytes (Fig. 5). This was confirmed with Trypan blue viable staining upon collagenase digestion of the fragments. The viability was consistently 95–98%. In *experiment I*, OCD-sourced constructs were less cellular (1.8 ± 0.6 cells per 1000 *μ*m^2^, 13.9 ± 2.7 μm mean cell diameter) than HAC-sourced constructs (2.6 ± 0.9 cells per 1,000 *μ*m^2^, 12.1 ± 2.5 μm diameter). Cellular diameters and overall cellularity were greater in the neocartilage than in the native tissue (1.4 ± 0.8 cells per 1000 *μ*m^2^, 9.5 ± 3.0 μm diameter) (Fig. 5). Cell distribution was more uniform in OCD-chon constructs. The H&E and SAF O/FG staining of HAC- and OCD-chon constructs appeared more intense than that of native tissue which may indicate a higher glycosaminoglycan (GAG) content in HAC- and OCD-chon constructs (Fig. 5). While Picrosirius Red (PSR) staining appeared more intense on the healthy articular cartilage sections than neocartilage, suggesting lower collagen content in HAC- and OCD-chon constructs (Fig. 5).

**Figure 6:**
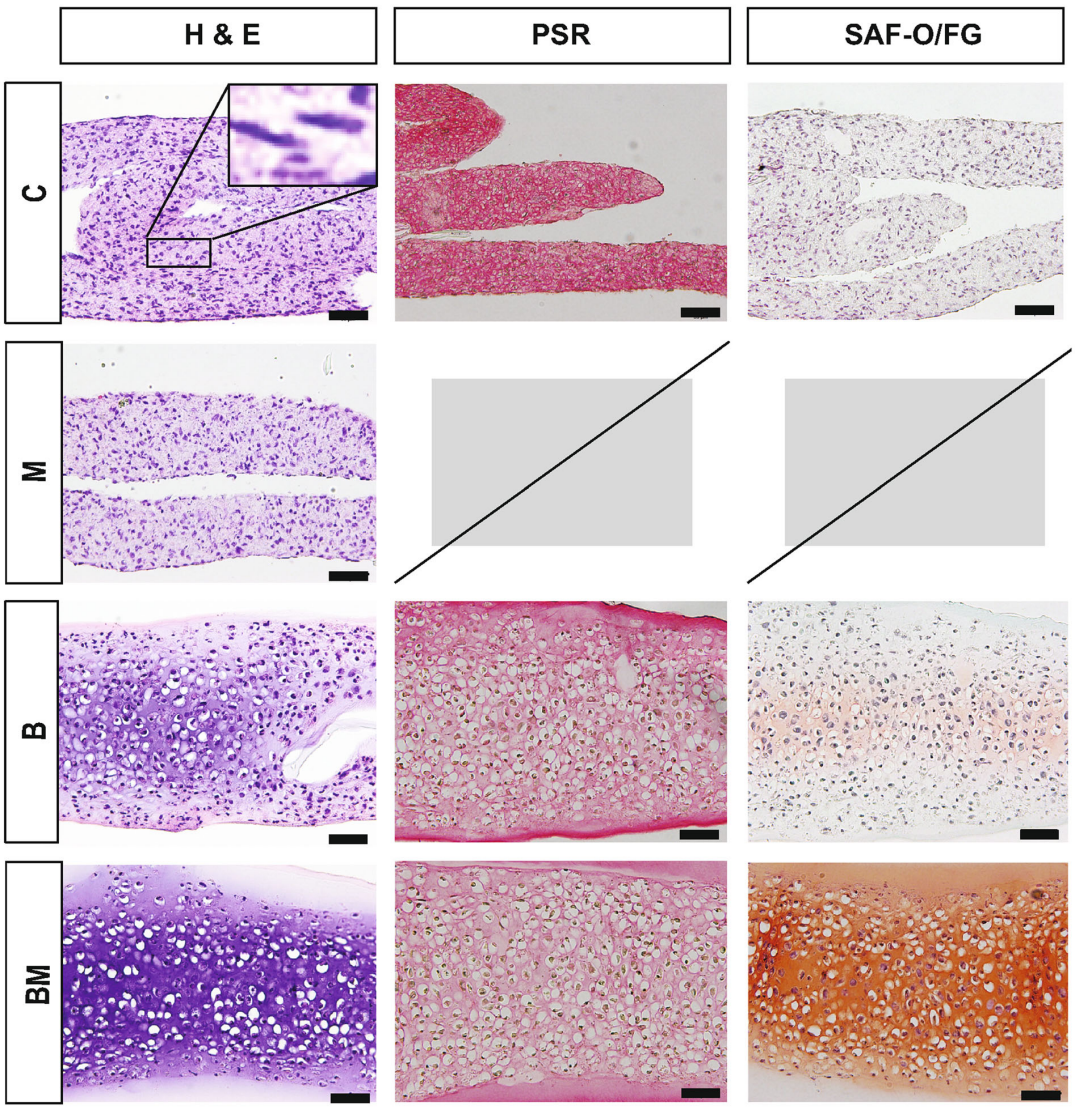
Histology of TE neocartilage constructs, Experiment II. Constructs in the control group subjected to culture media alone (C), Biological stimuli only (B), Mechanical stimuli only (M), Biological and mechanical stimuli combined (BM). Note high cellularity and spindle cell morphology of chondrocytes in groups C and M (inset). H & E = Hematoxylin and Eosin stain, PSR = Picrosirius Red, SAF O/FG = Safranin O/Fast green counterstain. Size bars = 50 *µ*m. Missing histology images are due to the small sample size.

Comprehensive histological assessment of constructs was impossible in *experiment II* due to a low number of constructs in some groups. H&E staining revealed differences in cell morphology and cellularity (Fig. 6). Specifically, cell morphology in the C and M groups was more fibroblastic (spindle cells) than chondrogenic, and cell density was markedly higher (C group: 6.3 ± 0.9 cells per 1000 *µ*m^2^; M group: 6.1 ± 1.6 cells per 1000 *µ*m^2^). In contrast, cells in B and BM groups had round-shaped cells resting in their lacunae, consistent with chondrocytic morphology, and comparable cell density between groups (B group: 4.6 ± 0.9 cells per 1000 *µ*m^2^; BM group: 4.0 ± 0.8 cells per 1000µm^2^). The staining intensity of the ECM on H&E was the greatest in the BM group, followed by the B group, M group, and C group.

**Figure 7:**
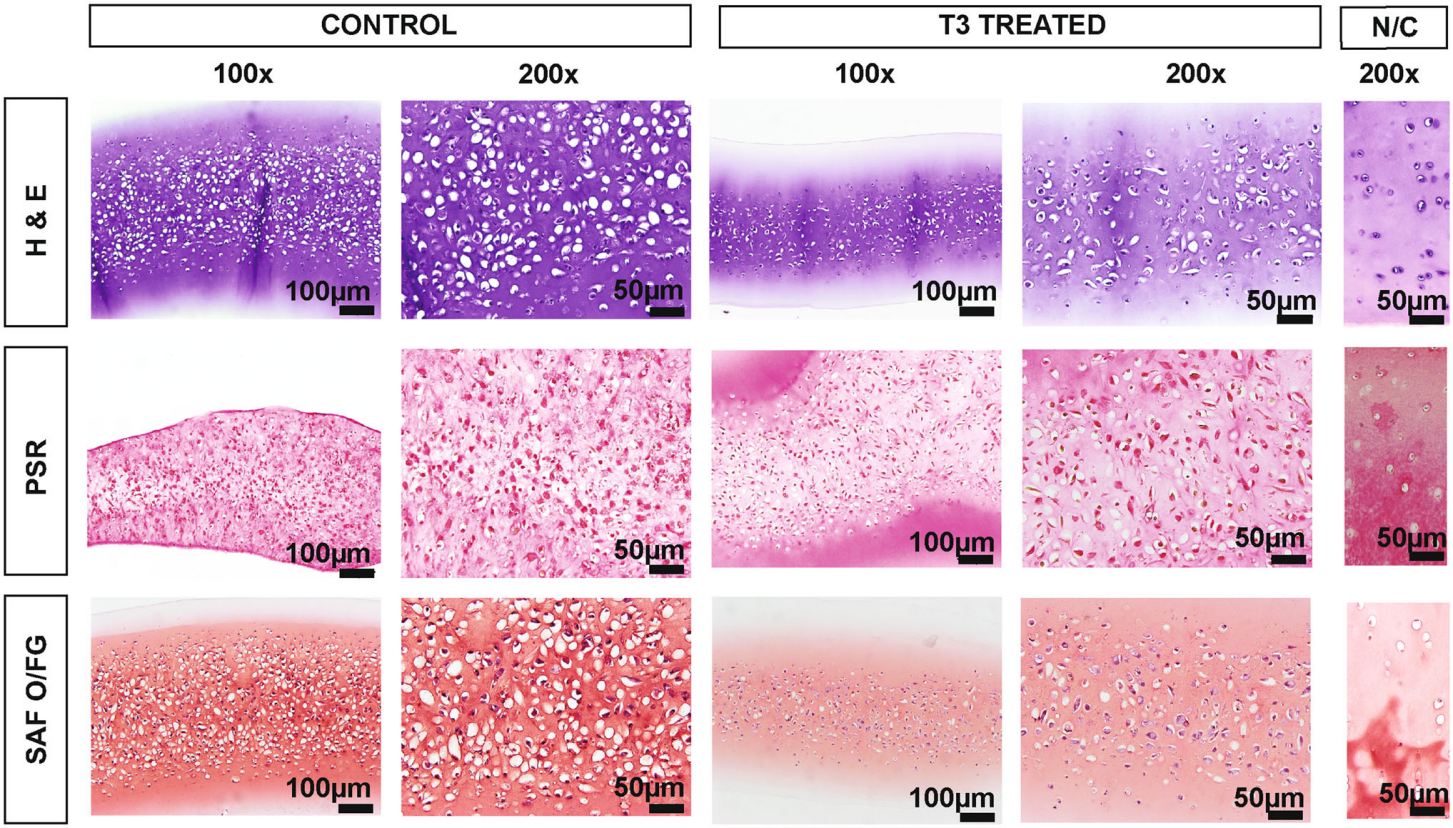
Histology of TE neocartilage constructs, Experiment III. T3-treated HAC chondrocyte-sourced TE neocartilage constructs (*n* = 11) and control TE neocartilage constructs (*n* = 11) compared to native articular cartilage tissue (N/C). *H & E* hematoxylin and Eosin stain, *PSR* picrosirius red, *SAF O/FG* Safranin O/Fast green counterstain. N/C is a staining control on healthy articular cartilage. Size bars at 100x = 100 *µ*m; 200x = 50 *µ*m.

*In experiment III*, the histomorphology of the constructs was similar to that observed in OCD and HAC constructs from experiment I and groups B and BM in experiment II, and was consistent with cartilage tissues (Fig. 7). The cellularity of constructs in the T3 group (2.9 ± 0.9 cells per 1000 *μ*m^2^) was lower than in the C group (3.1 ± 0.7 cells per 1000 *μ*m^2^). Cell diameters in T3 and control groups were 13.5 ± 2.7 *μ*m and 13.2 ± 3.0 μm, respectively, which is larger than native tissue (9.5 ± 3.0 *μ*m). Picrosirius red staining of T3 constructs appeared more intense than in control, while SAF O/FG was almost identical in intensity.

In all three experiments, the cell viability within the neocartilage constructs was confirmed by a board-certified histopathologist (NV). Also, no evidence of cell migration from neocartilage constructs was detected at any stage of the experiments.

### Biochemistry

**Figure 8:**
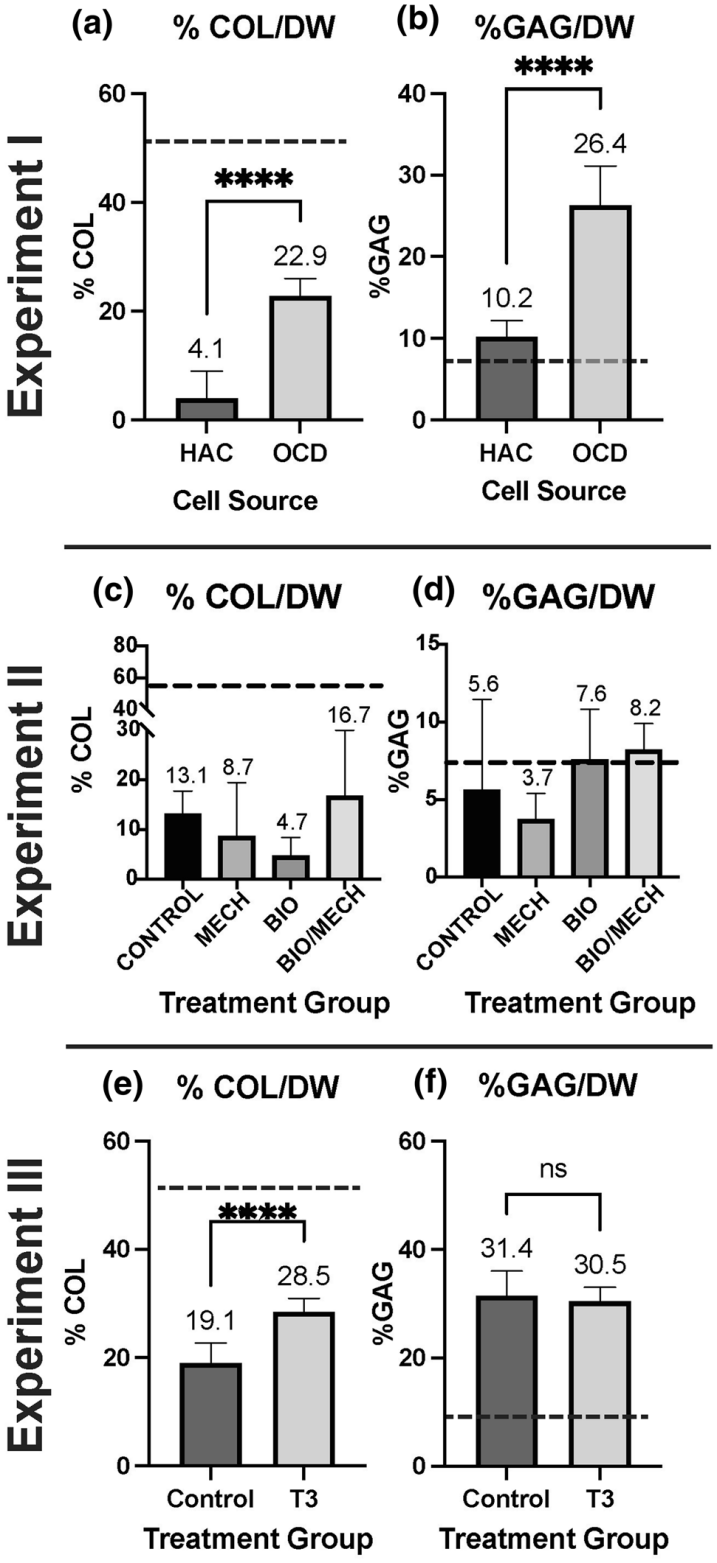
Biochemical properties of TE neocartilage constructs in Experiments I-III per dry weight (DW). *In experiment I*, HAC-chon-sourced TE neocartilage constructs (*n* = 8) compared to OCD-chon-sourced (*n* = 6). *In experiment II,* OCD-chon-sourced TE neocartilage constructs were exposed to different stimulation regimens. Control = minimal culture conditions, no growth factor or mechanical stimuli (*n* = 3); Mech = minimal culture conditions with mechanical stimulation only (*n* = 2); Bio = culture media was supplemented with TGF-β1, C-ABC, and LOXL2 (*n* = 9); Bio/mech = biological and mechanical stimuli combined (*n* = 6). *In experiment III*, HAC-chon-sourced TE neocartilage manufactured with (*n* = 11) and without (*n* = 10) T3 supplementation are compared. The numbers above charts represent the means of the reported values, and error bars correspond to standard deviation. The dashed line represents average reference values of native healthy articular cartilage in dogs with a median age of 3y. **** *p* < 0.0001; *** *p* < 0.001; ** *p* < 0.01; * *p* < 0.05; ns or no connecting lines= no significant difference.

Biochemical analyses supported histological observations. *In experiment I*, there was significantly more collagen in OCD-sourced constructs (*p* < 0.05, 22.9 ± 2.9% DW) than HAC constructs (4.1 ± 4.9% DW). However, relative to native tissue (51.1 ± 6.0% DW), both HAC-chon and OCD-chon TE constructs had lower collagen content per dry weight. (Fig. 8). GAG content was also significantly higher in OCD (26.4 ± 4.8%DW) than HAC constructs (10.2 ± 2% DW). In both OCD and HAC neocartilages, the GAG content was higher than in the native tissue (7.0 ± 3.3% DW).

No statistically significant differences were found in the biochemical properties between treatment groups in the multifactorial stimulation study (*experiment II)*. The BM group means trended higher in collagen content (16.7 ± 13.2 *µ*g/*µ*g DW) than B (4.7 ± 3.7 *µ*g/*µ*g DW), M (8.7 ± 10.8 *µ*g /*µ*g DW), and C (13.1 ± 4.7 *µ*g/*µ*g DW) groups. The BM group had higher GAG content (8.2 ± 1.7 *µ*g/*µ*g DW) than B (7.6 ± 3.3 *µ*g/*µ*g DW), M (3.8 ± 1.7 *µ*g/*µ*g DW), and C (5.62 ± 5.8 *µ*g/*µ*g DW) groups (Fig. 8). The GAG content in the B and BM groups was on par with native tissue.

Biochemical evaluation of constructs supplemented with T3 revealed significantly more collagen (28.5 ± 2.5 *µ*g/*µ*g DW) than in the C group (17.0 ± 7.7 *µ*g/*µ*g DW). On the contrary, there was no significant difference in GAG content between T3 (30.5 ± 2.5 *µ*g/*µ*g DW) and C (31.4 ± 4.6 *µ*g/*µ*g DW) constructs (Fig. 8).

### Mechanical Properties

**Figure 9:**
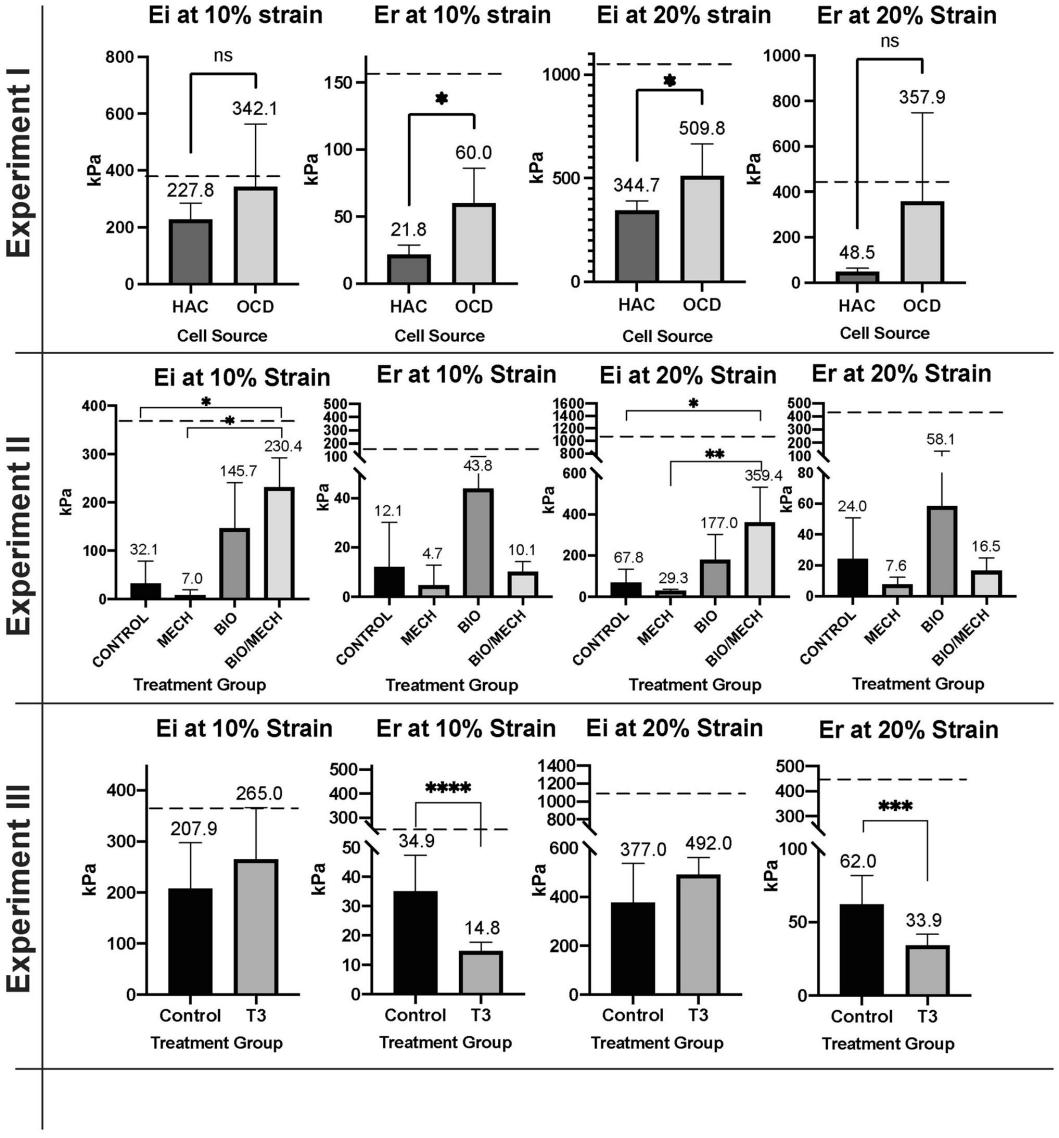
Compressive mechanical properties of TE neocartilage constructs in Experiments I-III. *In experiment I*, HAC-chon-sourced TE neocartilage constructs (*n* = 8) compared to OCD-chon sourced (*n* = 6). *In experiment II,* OCD-chon-sourced TE neocartilage constructs were exposed to different stimulation regimens. Control = minimal culture conditions, no growth factor or mechanical stimuli (*n* = 3); Mech = minimal culture conditions with mechanical stimulation only (*n* = 2); Bio = culture media was supplemented with TGF-β1, C-ABC, and LOXL2 (*n* = 9); Bio/mech = biological and mechanical stimuli combined (n=6). *In experiment III*, HAC-chon-sourced TE neocartilage manufactured with (*n* = 11) and without (*n* = 10) T3 supplementation are compared. The numbers above charts represent the means of the reported values, and error bars correspond to standard deviation. The dashed line represents average reference values of native healthy articular cartilage in dogs with a median age of 3y. Ei = instantaneous modulus, Er = relaxation modulus. 10% and 20% represent displacement (strain). **** *p* < 0.0001; *** *p* < 0.001; ** *p* < 0.01; * *p* < 0.05; ns or no connecting lines = no significant difference.

The instantaneous (Ei) and relaxation (Er) moduli of HAC-chon and OCD-chon sourced constructs were 227.8 ± 58.1 (Ei) and 21.8 ± 7.0 kPa (Er) and 342.1 ± 221.6 (Ei) and 60.0 ± 26.1 kPA (Er), respectively at 10% strain. At 20% strain, Ei and Er moduli of HAC-chon and OCD-chon sourced neocartilages were 344.7 ± 46.5 kPa (Ei) and 48.5 ± 15.0 kPa (Er) and 509.8 ± 155.3 kPA and 357.9 ± 391.3 kPA (Er) respectively **(**Fig. 9). Age-matched native tissue had an instantaneous (Ei) and relaxation (Er) moduli of 375.5 ± 145.4 kPa and 153.4 ± 50.0 kPa, respectively at 10% strain and 1082.9 ± 543.2 and 439.9 ± 216.6 kPA, respectively at 20% strain. OCD-chon sourced neocartilages were significantly stiffer between both cell sources than HAC-chon neocartilages at 20% strain and had significantly greater relaxation moduli at 10% strain.

In the multifactorial stimulation study (experiment II) we detected significantly higher instantaneous compressive stiffness values (Ei) in B (145.7 ± 95.6 kPa) and BM (230.4 ± 62.4 kPa) groups than the C (32.1 ± 46.5 kPa) and M (7.0 ± 12.2 kPa) at 10% strain. At 20% strain, we noted a similar trend in our results with B (177.0 ± 126.8 kPa) and BM (359.4 ± 173.9 kPa) groups having higher values than the C (67.76 ± 65.4 kPa) and M (29.32 ± 7.4 kPa) (Fig. 9).

T3 supplementation in experiment III also resulted in higher compressive stiffness of the constructs with instantaneous moduli of 265 ± 101.3 MPa at 10% strain as compared to 207.9 ± 89.9 MPa in controls. This difference was not statistically significant. A similar trend in compressive stiffness was observed at 20% displacement. An inverse effect was detected in relaxation moduli. T3 supplementation resulted in significantly lower relaxation moduli (*p* < 0.01) at 10% displacement of TE constructs (14.8 ± 2.9 MPa) than controls (34.9 ± 12.5 MPa) (Fig. 9).

**Figure 10:**
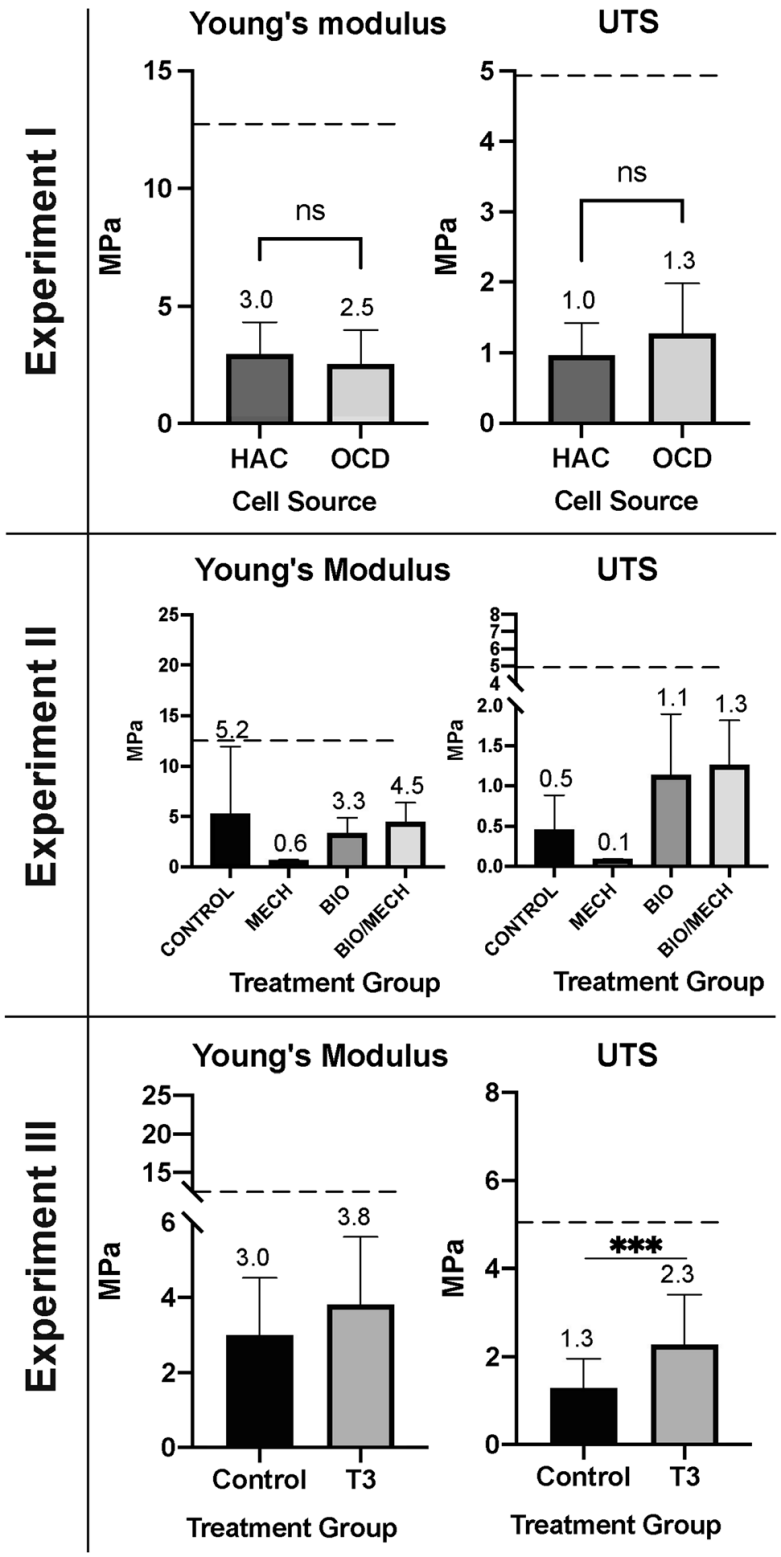
Tensile mechanical properties of TE neocartilage constructs in Experiments I-III. *In experiment I*, HAC-chon-sourced TE neocartilage constructs (*n* = 8) compared to OCD-chon-sourced (*n* = 6). *In experiment II,* OCD-chon-sourced TE neocartilage constructs were exposed to different stimulation regimens. Control = minimal culture conditions, no growth factor or mechanical stimuli (*n* = 3); Mech = minimal culture conditions with mechanical stimulation only (*n* = 2); Bio = culture media was supplemented with TGF-β1, C-ABC, and LOXL2 (*n* = 9); Bio/mech = biological and mechanical stimuli combined (*n* = 6). *In experiment III*, HAC-chon-sourced TE neocartilage manufactured with (*n* = 11) and without (*n* = 10) T3 supplementation are compared. The numbers above charts represent the means of the reported values, and error bars correspond to standard deviation. The dashed line represents average reference values of native healthy articular cartilage in dogs with a median age of 3y. UTS = ultimate tensile strength. Young’s moduli represent tensile stiffness. **** *p* < 0.0001; *** *p* < 0.001; ** *p* < 0.01; * *p* < 0.05; ns or no connecting lines= no significant difference.

Tensile stiffness (Young’s moduli) of HAC-chon and OCD-chon sourced constructs was 3.0 ± 1.45 MPa and 2.6 ± 1.45 MPa, respectively (Fig. 10). The tensile strength (UTS) was 1.0 ± 0.5 MPa and 1.3 ± 0.7 MPa, respectively. There was no statistically significant difference between HAC-chon and OCD-chon groups, however the OCD-chon construct means trended stronger than HAC-chon. Relative to native tissue (Young’s: 13.2 MPa, UTS: 5.0 MPa), constructs from both groups were, less stiff and less strong.

Following multifactorial stimulation (experiment II), we found that tensile stiffness (Young’s moduli) of BM (4.5 ± 1.9M Pa) and C (5.22 ± 6.7 MPa) groups trended slightly higher than B (3.3 ± 1.6 MPa) and much higher than M (0.6 ± 0.1 MPa) groups, but these differences were not significant statistically. Tensile strength of the constructs (UTS) was the greatest for BM (1.3 ± 0.6 MPa) and B (1.1 ± 0.8 MPa) groups, lower for the C (0.5 ± 0.4 MPa), and lowest for M (0.1 ± 0.004 MPa) groups (Fig. 10).

T3 supplementation in experiment III, resulted in significantly higher UTS of neocartilage constructs (2.3 ± 1.1 MPa) over controls (1.3 ± 0.7 MPa) (*p* < 0.05). Tensile stiffness (Young’s moduli) was higher in the T3 group (3.8 ± 1.8 MPa), but not significantly greater than in the C group (3.0 ± 1.5 MPa) (Fig. 10).

### Immunohistochemistry

**Figure 11:**
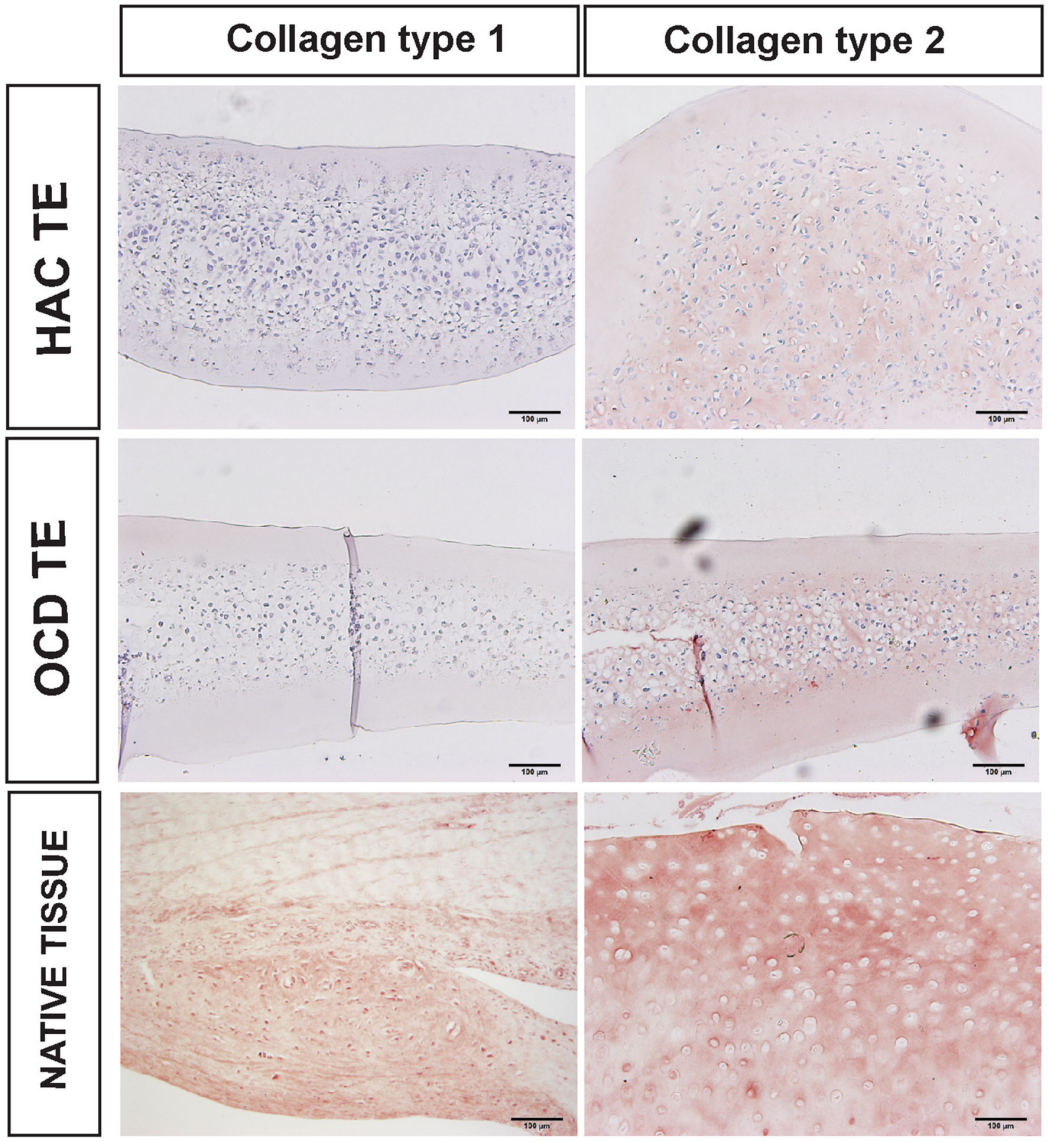
Immunohistochemical analysis for collagens type I, II. The positive tissue controls for collagen I, II canine tendon and articular cartilage, respectively. Size bar = 100 *µ*m.

**Figure 12:**
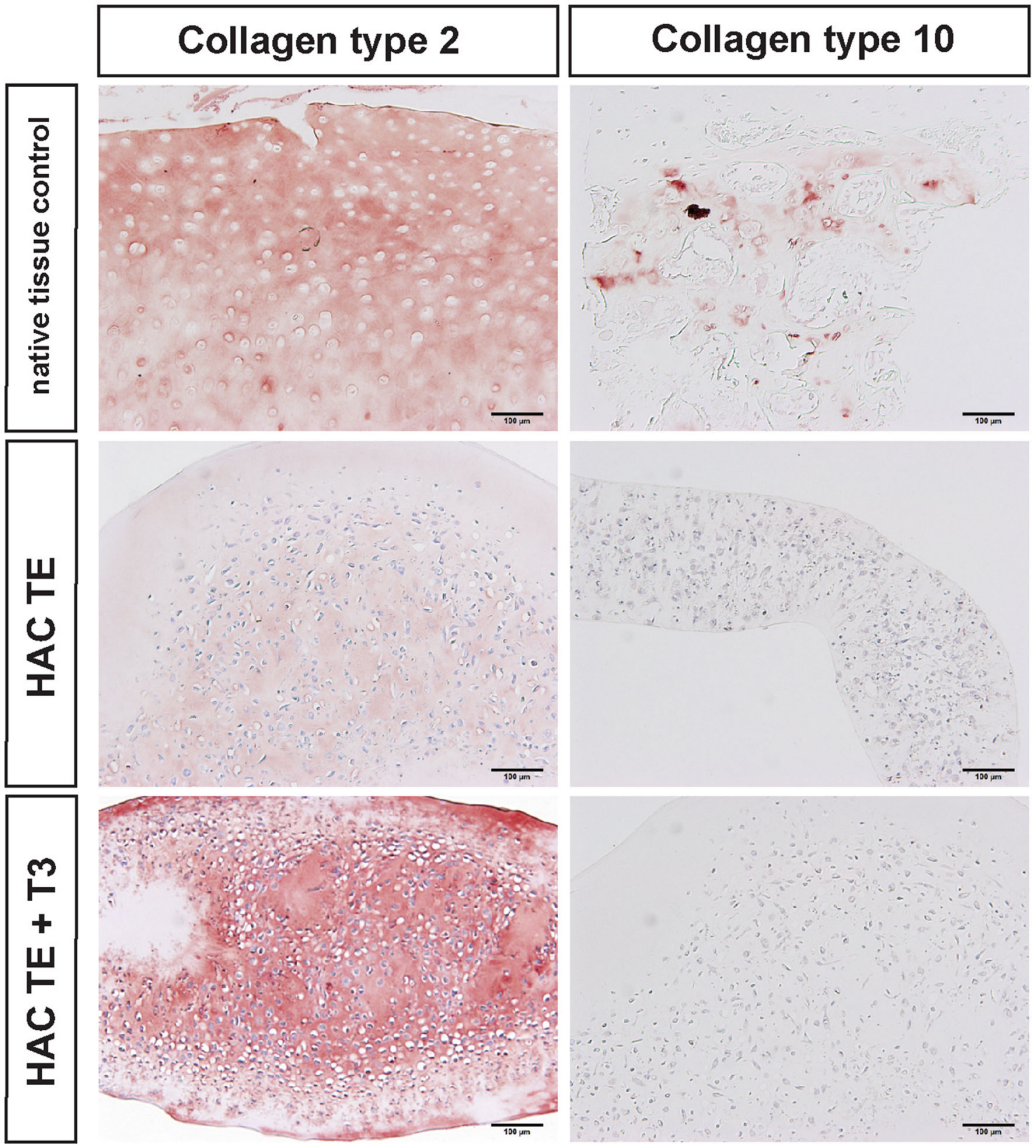
Immunohistochemical analysis for collagen type II and X. The positive controls for collagen II and X are canine articular cartilage and growth plate, respectively. Size bar = 100 *µ*m.

Immunohistochemical screening for the presence and distribution of collagens Type I, II, and X revealed an overall consistent absence of collagen type I in the TE constructs (Figs. 11 and 12). Because the size of the cells trended higher in the TE constructs relative to native tissue, immunohistochemical analysis for collagen type X was conducted to rule out hypertrophy. The results point out that T3 supplementation did not result in the deposition of collagen type X. Due to a lack of testable tissue, we were unable to confirm this statement in experiment II.

### Donor Comparison

**Figure 13:**
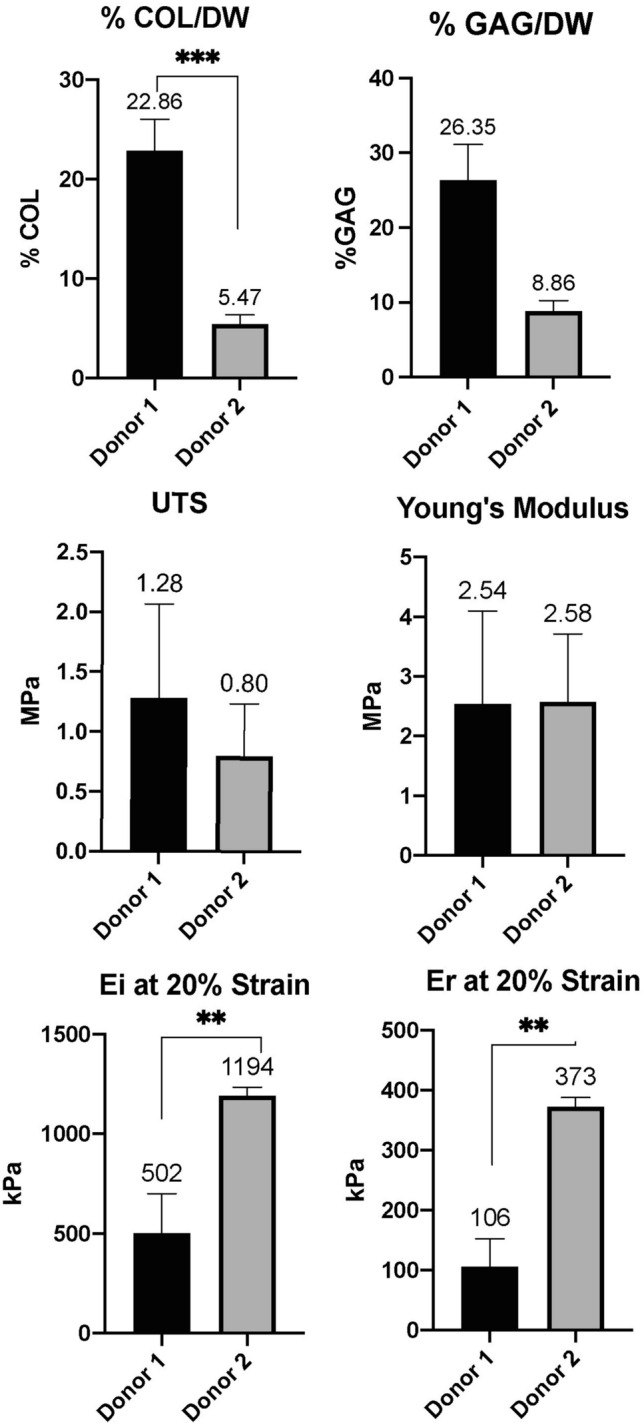
TE construct properties between two OCD chondrocyte donors. Donor 1 (*n* = 6), Donor 2 (*n* = 2). Numbers above the columns represent the means. Ei = instantaneous modulus, Er = relaxation modulus. 20% represent displacement (strain). *** *p* < 0.001; ** *p* < 0.01; * *p* < 0.05.

We detected a significant difference in properties of TE neocartilage constructs engineered using identical methods between two donors (Fig. 13). Specifically, constructs from Donor 1 (shown in experiment I) had significantly more collagen and notably more GAG. These differences had no effect on the tensile properties of the constructs as no significant difference was detected between the two in UTS and Young’s modulus. On the contrary, the compressive stiffness of the Donor 2 was greater than that of Donor 1 despite lower GAG and collagen content.

## Discussion

This study is the first to demonstrate that chondrocytes derived from OCD cartilage fragments can be used to engineer scaffold-free neocartilage tissue. We found that chondrocytes isolated from OCD surgery fragments could form functional cartilage constructs with properties comparable to constructs engineered from healthy articular chondrocytes. This indicates that chondrocytes isolated from OCD flaps are not inferior to healthy articular chondrocytes for neocartilage engineering. Although biochemical and tensile properties of OCD and HAC sourced TE constructs were inferior to native tissue, the compressive properties of TE constructs from both cell sources at 10% strain approached properties of native tissue of the same joint. The second and third experiments focused on improving the mechanical and biochemical properties of tissue-engineered cartilage constructs by applying mechanical and biological stimuli that were shown effective in other animal species. In experiment II, the addition of TGFβ-1, C-ABC, LOX-L2, and mechanical stimulation during the construct maturation period, improved the biomechanical properties of neocartilages generated from OCD chondrocytes. In the third experiment, we determined that the addition of tri-iodothyronine increased the collagen content in HAC neocartilages 1.5-fold (33%), which is 1.8-times (44%) less than native tissue. Collectively, the data suggest that OCD chondrocytes can serve as a potential cell source for cartilage TE and that canine chondrocytes respond favorably to biological and mechanical stimuli shown effective in other animal species.

The gross morphology of TE neocartilage constructs was very similar between HAC and OCD cell sources as long as TGFβ-1 was supplemented (experiment I, experiment II groups B and BM, and experiment III). Also, the overall thickness of the neocartilages regardless of cell source (0.5–0.7mm) was comparable to the thickness of native articular cartage in canine species (0.56 mm) (Fig. 4). The histomorphological examination and viability assessment of OCD chondrocytes revealed that the majority of chondrocytes are viable (Fig. 4). The presence of viable chondrocytes in OCD flaps has been reported in humans^3^ and in dogs.^15,24^ However, to the best of our knowledge, OCD chondrocytes have not been utilized for TE of neocartilage before. OCD chondrocytes grew well *in vitro* and had comparable population kinetics to HAC chondrocytes (Fig. 2). As with HAC chondrocytes, aggregate redifferentiation facilitated chondrogenic phenotype in OCD cells, based on their ability to self-assemble into cartilaginous constructs. Real-time PCR analyses revealed no significant difference in Aggrecan, Collagen type I, II, and X gene expression between OCD and HAC chondrocytes, which further supports the consideration of OCD chondrocytes for neocartilage tissue-engineering.

On histology, OCD-chon and HAC-chon-sourced TE constructs were 1.2- and 1.8-times (22% and 46%) more cellular than native tissue, and cell diameter was 1.4- (36%) and 1.2-times (21%) larger than the native counterpart (Figs. 5, 6, and 7). The discrepancy in cell size between native and engineered cartilage aligns with reports by Lee et al., who showed similar cell morphology and distribution in cartilage constructs engineered from bovine primary chondrocytes using the scaffold-free method.^29^ The cell size and the cellularity of the bovine TE neocartilage constructs decreased following implantation *in vivo*.^29^ It is possible that larger cell size is due to the higher synthetic activity of chondrocytes in the neocartilage, which progressively normalizes as the tissue matures and receives mechanical stimulation.^14,36^ The decrease in construct cellularity also could be attributed to developmental changes that accompany native cartilage maturation,^53^ where overall cellularity of tissue decreases with age. Yet, the amount of ECM does not increase but changes its composition. Specifically, the collagen network is stabilizing via the formation of pyridinoline crosslinks, which, in turn, increases the tensile stiffness and strength of the cartilage.^53^ GAG composition is also changing, with the ratio of chondroitin sulfate to keratan sulfate decreasing with maturation.^53^ For tissue architecture, cells become organized in a more specific pattern in the adult.

HAC-chon and OCD-chon TE constructs had 1.5- (30%) and 3.7- times (73%) more GAG than native tissue, respectively. On the contrary, the collagen content was 12.5- (91%) and 2.2-times (55%) less than native tissue (Fig. 8). These observations agree with work by Huwe et al. where neocartilage was generated from costal chondrocytes using a scaffold-free method.^20^ These constructs contained ~1.25-times more GAG and ~5-times less collagen than the native bovine articular cartilage.^20^ As with cellularity, GAG content may reflect the immature nature of the neocartilage. Studies done by Elliot et al. demonstrated that GAG content per dry weight decreases from 50% at birth to 15% in adulthood.^9^ Furthermore, one study comparing the GAG content of neocartilage prior and following implantation into TMJ disc showed that within six weeks following orthotopic implantation, the GAG content of neocartilage decreased by at least 4-times and assumed concentration akin to native tissue.^48^ These findings collectively suggest that, similarly to native tissue, engineered neocartilage may undergo a maturation process represented by shifts in cellularity and extracellular matrix composition. Perhaps, fetal articular cartilage would have been a better native control tissue for this experiment.

OCD-chon sourced neocartilages outperformed HAC-chon-sourced in terms of their GAG and collagen content. This is interesting because no difference in aggrecan or Collagen type I and II gene expression was detected. Collectively, these findings invite further investigation, especially in light of one of the proposed theories for OCD pathogenesis that suggests abnormal collagen metabolism.^27,38^ It is possible that OCD chondrocytes are primed by the proinflammatory mediators present in the OCD joint to produce more collagen. Indeed, Clements et al. showed that canine chondrocytes removed from an osteoarthritic environment had upregulation of collagen type II gene expression.^51^ An increase in collagen synthesis could represent a mechanism of self-repair that has been proposed in OCD literature.^27^ Since no increase in tensile stiffness or strength was associated with higher collagen content in OCD-chon-sourced constructs, other elements, such as collagen crosslinking, may play a role and warrant further investigation. The structure and organization of collagen crosslinks undergo various changes throughout hyaline cartilage maturation in an animal.

Although no significance was detected, there was an obvious trend of improvement in collagen and GAG content with an application of biochemical stimulation during neocartilage maturation in experiment II. These finding echo the outcomes of the study performed by Kwon et al., who applied similar biological stimuli on human chondrocytes, and underscore the advantage and importance of identifying the optimal regimen for the application of C-ABC and LOXL2.^25^ To bring the collagen content in the TE constructs on par with native tissue, we explored the effects of tri-iodothyronine (T3) supplementation on the biomechanical properties of HAC-chon sourced TE neocartilage constructs. A significant increase (1.5-fold, 33%) in total collagen content in the T3 supplemented group over the control was found. This experiment confirmed that canine chondrocytes, just like rabbit and bovine cells^28,52^ respond to T3 supplementation by the increase in collagen content in the ECM.

In terms of biomechanics, HAC-chon and OCD-chon-sourced TE constructs were 3.1-times (68%) and 2.1-times (52%) less stiff under compression at 20% strain than native canine cartilage. Compared to studies using a similar scaffold-free tissue engineering strategy, these properties translate well to other species. For instance, Huwe *et al*., who engineered cartilage from porcine costal chondrocytes, demonstrated a similar discrepancy in mechanical properties when engineered constructs were compared to native bovine articular cartilage from the knee.^20^ Also, Kwon et al. utilized human articular chondrocytes to engineer cartilage and reported similar mechanical compressive stiffness (− 170 kPA) of the engineered cartilage to properties reported here.^25^ Of note, these properties were improved significantly after additional biological stimuli, such as LOXL1 and C-ABC, were applied.^25^ Indeed, in our experiment II addition of C-ABC and LOXL2 to the biological stimuli showed benefit in improving the compressive properties of OCD-chon sourced neocartilage.

In experiment II, the supplementation of TGFβ-1, LOXL1, C-ABC, and static mechanical compression stimulation showed a significant improvement in the compressive properties of neocartilage constructs compared to controls. Similar observations were reported by Huwe and Sullan *et al*. study. The synergistic effects of biological stimuli (TGF-β1, LOXL2, and C-ABC) with mechanical static compression were observed on the neocartilages manufactured from porcine costal chondrocytes.^22^ The potential explanation of the effects of factors is the facilitation of the anabolism and production od ECM molecules by TGFβ-1, reduction of GAG by C-ABS, and subsequent formation of pyridinoline cross links by LOX L2.

The results of experiment II indicate that canine chondrocytes respond to biological and mechanical stimuli similarly to chondrocytes from other species.^21,25,33^ Further studies investigating the effects of each stimulus independently in OCD and HAC neocartilage constructs will benefit both human and canine patients as we progress toward the development of novel cartilage replacement therapies.

Under tension, HAC-chon and OCD-chon-sourced TE constructs had 20% and 26% strength of native tissue, respectively, but there was no significant difference in tensile strength or tensile stiffness between OCD and HAC-chon-sourced TE constructs. Supplementation of T3 in experiment III increased the collagen content in the neocartilage, leading to improvement of tensile strength and stiffness. With T3 supplementation, the tensile strength improved significantly and comprised 46% of native tissue (Fig. 9), i.e., 26% improvement. Lee *et al*. demonstrated very similar tensile properties on neocartilage manufactured from juvenile bovine chondrocytes, where ~ 60% improvement in tensile strength was achieved with T3 supplementation.^28^ Given that T3 is known to stimulate chondrocyte hypertrophy and deposition of collagen type X,^43^ immunohistochemistry was performed to identify collagen types within the TE constructs. The IHC revealed no evidence of collagen type X deposition in the T3 treated or control constructs. On the contrary, greater intensity of collagen type II immunolabeling in the T3 treated constructs was detected (Figs. 11 and 12). One limitation of the thyroid stimulation experiment is it was performed on HAC-sourced chondrocytes and not OCD-sourced. It would be important to perform a similar study and compare the effects of thyroid supplementation on HAC vs. OCD-sourced neocartilage constructs. Furthermore, it would also be imperative to combine thyroid supplementation with other biological stimuli and mechanical compression.

Donor comparison revealed significant differences in biomechanical properties of constructs (Fig. 13). The reason for this discrepancy could be due to individual donor variability and potential differences in pathogenesis, treatment, and duration of the OCD. It is also possible that the low number of constructs for Donor 2 had an effect on this outcome, and larger numbers of constructs from a range of donors need to be compared in future studies. It would be important to compare the properties of cartilage among the OCD flaps before the cells were extracted and expanded. For instance, the duration of the OCD may influence gene expression and subsequent performance of chondrocytes in culture. In this study, Donor 1 was a 9-month-old border collie and donor two was a 10-month-old German shorthair pointer. Although the ages of the two donors were similar, the duration of clinically manifested OCD was not. Donor 1 had the surgery done 3.5 months after the initial diagnosis, and donor 2 had surgery performed 1.5 months after the initial diagnosis. Careful comparison and analyses of the OCD flaps would be important to determine what markers can predict positive outcomes of TE. Furthermore, prospective experiments comparing gene expression in chondrocytes and properties of TE constructs sourced from various OCD donors may shed light on this condition’s pathogenesis.

Nevertheless, the advantages of our approach can provide readily available source of cryopreserved autologous chondrocytes for autologous and possible also allogeneic applications. OCD patients sometimes return to clinics with a similar problem in other joints and thus can greatly benefit from having their own cells available for regenerative applications. Furthermore, when carefully examining the current pathogenesis studies of OCD, the core of the problem is the vascularization of the cartilage-bone interface during epiphyseal chondrogenesis and not in the chondrocytes.^5,41^ Thus, the majority of chondrocytes in the OCD flaps are actually viable.^3,4^ They may have altered gene expression due to the absence of proper mechanical stimulation and osteoarthritis-induced changes, but this is not a permanent change. In support of the latter, our gene analyses reveled no difference in aggrecan, collagen I, II, and X gene expression between OCD and HAC chondrocytes.

In summary, this study is the first to explore the potential utility of OCD fragments removed and discarded during routine OCD surgery in canine patients as a novel cell source for neocartilage TE. Future research should be done identifying the role of the duration of OCD lesion on the fitness of these chondrocytes for TE. This study also underscored the potential importance of thyroid hormone in the chondrocyte metabolism that warrants further investigation.
